# Weighted bootstrapping: a correction method for assessing the robustness of phylogenetic trees

**DOI:** 10.1186/1471-2148-10-250

**Published:** 2010-08-17

**Authors:** Vladimir Makarenkov, Alix Boc, Jingxin Xie, Pedro Peres-Neto, François-Joseph Lapointe, Pierre Legendre

**Affiliations:** 1 Département d'informatique, Université du Québec à Montréal, C.P. 8888 succ. Centre-Ville, Montreal (QC) H3C 3P8 Canada; 2 Département de sciences biologiques, Université du Québec à Montréal, C.P. 8888 succ. Centre-Ville, Montreal (QC) H3C 3P8 Canada; 3 Département de sciences biologiques, Université de Montréal, C.P. 6128 succ. Centre-Ville, Montréal, Québec, H3C 3J7 Canada

## Abstract

**Background:**

Non-parametric bootstrapping is a widely-used statistical procedure for assessing confidence of model parameters based on the empirical distribution of the observed data [1] and, as such, it has become a common method for assessing tree confidence in phylogenetics [2]. Traditional non-parametric bootstrapping does not weigh each tree inferred from resampled (i.e., pseudo-replicated) sequences. Hence, the *quality *of these trees is not taken into account when computing bootstrap scores associated with the clades of the original phylogeny. As a consequence, traditionally, the trees with different bootstrap support or those providing a different fit to the corresponding pseudo-replicated sequences (the fit quality can be expressed through the LS, ML or parsimony score) contribute in the same way to the computation of the bootstrap support of the original phylogeny.

**Results:**

In this article, we discuss the idea of applying weighted bootstrapping to phylogenetic reconstruction by weighting each phylogeny inferred from resampled sequences. Tree weights can be based either on the least-squares (LS) tree estimate or on the average secondary bootstrap score (SBS) associated with each resampled tree. *Secondary bootstrapping *consists of the estimation of bootstrap scores of the trees inferred from resampled data. The LS and SBS-based bootstrapping procedures were designed to take into account the quality of each "pseudo-replicated" phylogeny in the final tree estimation. A simulation study was carried out to evaluate the performances of the five weighting strategies which are as follows: LS and SBS-based bootstrapping, LS and SBS-based bootstrapping with data normalization and the traditional unweighted bootstrapping.

**Conclusions:**

The simulations conducted with two real data sets and the five weighting strategies suggest that the SBS-based bootstrapping with the data normalization usually exhibits larger bootstrap scores and a higher robustness compared to the four other competing strategies, including the traditional bootstrapping. The high robustness of the normalized SBS could be particularly useful in situations where observed sequences have been affected by noise or have undergone massive insertion or deletion events. The results provided by the four other strategies were very similar regardless the noise level, thus also demonstrating the stability of the traditional bootstrapping method.

## Background

In statistics, bootstrapping is a general purpose parameter estimation approach falling within a broader class of resampling methods [1]. Bootstrapping allows one to assess whether the data distribution has been influenced by stochastic effects. Non-parametric bootstrapping proceeds by generating pseudo-replicates of the observed data. Each of the pseudo-replicated data sets is obtained by random sampling with replacement from the original data set. On the other hand, parametric bootstrapping involves sampling from a fitted parametric model, obtained by substituting the maximum likelihood estimator for the unknown population parameter.

Non-parametric bootstrapping is the most commonly used robustness estimation method in phylogenetics [2,3]. It is applied to evaluate the reliability of a phylogenetic tree by examining how often a particular clade, or the corresponding branch, in the tree appears when the original nucleotides or amino acids are resampled. The tree inferring method used to reconstruct the phylogeny from the original data should be carried out to infer the phylogenies from the resampled data. The frequency with which a given branch is found represents its bootstrap support (i.e., bootstrap score).

Different parametric bootstrapping procedures related to phylogenetic analysis were proposed by Huelsenbeck et al. [4], Swofford et al. [5] and Goldman et al. [6]. Parametric bootstrapping can be carried out when we assume an explicit model of sequence evolution. In this case, the original data are used to estimate the stochastic evolutionary parameters, which may include the site-specific rates of evolution, the distribution from which the rates of evolution are drawn or the substitution probabilities on each branch, characterizing the original data set.

In spite of concerns, controversy and confusion over the interpretation of bootstrap scores [7-10], bootstrap analysis has been playing a prominent role in many phylogenetic studies and will likely remain a key method for assessing branch support of phylogenetic trees [11]. It is often assumed, for instance, that the bootstrap support of a branch represents the probability that this branch is correct. However, this point of view is over-simplified [12]. For example, in the case of the famous *Felsenstein zone quartet tree *the maximum parsimony and UPGMA methods converge to the wrong tree as the sequence length increases, and thus both assign very high bootstrap scores to the clades of the wrong phylogeny [13,14]. The best way to interpret the bootstrap support of a given clade is to consider that it indicates the probability that this clade would continue to be found if the same phylogenetic inferring method was applied to pseudo-replicated data having the same empirical distribution as the original data set [12].

In this article we introduce two weighting schemes which can be used to assign weights to each of the trees obtained from pseudo-replicated data. One of them is based on the LS estimate of "pseudo-replicated" trees, whereas the second one proceeds by assessing bootstrap support of those trees (i.e., carries out secondary bootstrapping). These two weighting schemes can be used to correct the standard non-parametric bootstrapping procedure that assign equal weights to each of the phylogenies obtained from the pseudo-replicated sequences. Such a correction will take into account *the quality *of each pseudo-replicated phylogeny. The LS coefficient, as well as the ML function value or the Maximum parsimony score, can be used as an estimate of how close the distance matrix obtained from the pseudo-replicated sequences (or the set of pseudo-replicated sequences, in the case of ML or MP) is to the space of trees. For instance, if it is located far away from this space (i.e., this corresponds to a high value of the LS coefficient) compared to the other trees inferred form pseudo-replicates, then a low weight should be assigned to this tree (and to this pseudo-replicated data set). Alternatively, secondary bootstrapping can be performed to obtain a *robustness estimate *for each of the trees built from pseudo-replicates. Each of the pseudo-replicated multiple sequence alignments (PRA) obtained from the original data can be resampled once again to obtain secondary pseudo-replicated multiple sequence alignments (SPRA) that can be, in turn, used to assess the bootstrap support of the tree inferred from PRA. In this way, an average bootstrap score of internal branches of each pseudo-replicated tree can be used to assign a weight to this tree. Thus, a higher average bootstrap score of a "pseudo-replicated" tree will correspond to a higher weight assigned to this tree.

This article is organized as follows. In the Methods section, we present two weighting schemes, based on the LS and secondary bootstrapping, used to assign weights to "pseudo-replicated" trees. There we also discuss the possibility of normalization of the obtained tree estimates. Then, in the Results section, we present the simulation results for the traditional (unweighted) bootstrapping and four different bootstrapping procedures inducing weights, while considering two real data sets of 12 DNA sequences (*Primate data set *from [15]) and 32 protein sequences (*PheRS sequences *from [16]). In these simulations, we also compare the robustness of the competing bootstrapping procedures by assessing their performances under the condition when different amounts of noise were added to the original data. The Discussion section compares the proposed methods with standard bootstrap correction procedures and explains the rationale of our study. Finally, the Conclusion section summarizes the introduced weighting schemes and presents the ideas for future research.

## Methods

Here we discuss four new weighting schemes which can be used in bootstrapping to assign weights to the trees obtained from pseudo-replicated sequences. Specifically, the LS (least-squares) and secondary bootstrap score estimates will be computed for each pseudo-replicated phylogeny. The normalized LS and normalized secondary bootstrap score estimates will be also considered. All these estimates can be used to generate weights of pseudo-replicated trees. A "corrected" bootstrapping procedure based on the obtained weights will be presented.

Let *X *be a set of *n *taxa (i.e., objects, species) and *A *be a multiple sequence alignment obtained for the taxa from *X*. We assume that each sequence in *A *has *l *nucleotides (or amino acids). The model of nucleotide substitution that best fits the data can then be determined and the corresponding data correction applied. A phylogenetic tree *T *can be inferred by a tree-building algorithm (the Neighbor-Joining [17] algorithm was used in this study to infer phylogenies). The standard non-parametric bootstrap scores can be calculated using the following procedure [2]:

(1) *l *columns of *A *are randomly chosen with replacements, giving rise to a pseudo-replicated sequence alignment *PRA *with *n *rows of *l *columns. This procedure is repeated *N *times and a set of pseudo-replicated sequence alignments *PRA*_1_, *PRA*_2_,..., *PRA*_*N *_is obtained.

(2) Phylogenetic trees *T*_1_, *T*_2_,..., and *T*_*N *_are then reconstructed from the pseudo-replicated alignments *PRA*_1_, *PRA*_2_,..., and *PRA*_*N*_, by means of the same tree-inferring algorithm that was used to build *T*.

(3) The topology of the original tree *T *is then compared to the topologies of the trees built from pseudo-replicates. The bootstrap score of the branch *k *in *T *(denoted here as *bs*_*k*_) is the percentage of time that *k *is found in the set of trees *T*_1_, *T*_2_,..., and *T*_*N*_. It is computed as follows:(1)

where *B*_*i *_is the set of internal branches of the tree *T*_*i*_, given by their non-trivial splits or bipartitions.

### LS-based bootstrapping

The least-squares (LS) coefficient can be used to estimate how well the given distance matrix **D**, obtained from the multiple sequence alignment *A *using a specific sequence-to-distance transformation, approximates the patristic distance (i.e., additive distance or tree metric) **Δ **between the leaves of the phylogenetic tree *T *obtained from **D **using the selected tree-building algorithm. In this study, the Jukes-Cantor distance [18] for the DNA sequences and Kimura Protein distance [19] for the amino acids were employed. The least-squares coefficient, *LS*, between **D **and **Δ **is computed as follows:(2)

where *d*(*i*,*j*) is the distance between the taxa *i *and *j*, and *δ*(*i*,*j*) is the patristic distance between the leaves labelled by *i *and *j *in the phylogenetic tree *T*.

**Figure 1:**
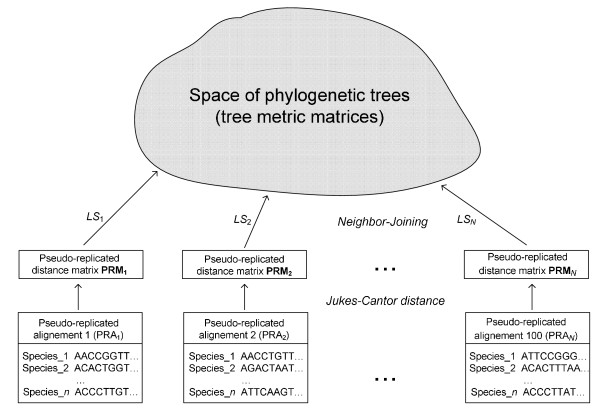
**LS-based bootstrapping**. LS-based bootstrapping: The LS coefficient can be used to assess the quality of phylogenetic trees obtained from pseudo-replicated sequence alignments. Lower values of LS correspond to a better fit by a phylogenetic tree and are associated with higher weights.

We propose to use the LS coefficient to assign individual weights to all trees obtained from pseudo-replicated data (Figure 1). Obviously, the smaller the value of the LS coefficient, the better the phylogenetic tree fits the corresponding distance matrix **D**. Instead of using equal weights for all trees obtained from pseudo-replicated data, as the traditional bootstrapping does, the following four-step weighting scheme was adopted in this study:

1. Given the original sequence alignment *A*, we first computed from it a series of *N *pseudo-replicated alignments *PRA*_1_, *PRA*_2_,..., and *PRA*_*N*_, using the traditional bootstrapping strategy. The Jukes-Cantor [18] evolutionary model was then applied to obtain the distance matrices **M**, **PRM**_1_, **PRM**_2_,..., and **PRM**_*N*_, from *A*, *PRA*_1_, *PRA*_2_,..., and *PRA*_*N*_, respectively. Phylogenetic trees *T*, *T*_1_, *T*_2_,..., and *T*_*N *_and the corresponding tree metric matrices **Δ**, **Δ**_1_, **Δ**_2_,..., and **Δ**_*N *_were calculated from these distance matrices using Neighbor Joining [17].

2. The vector **ls **= {*ls*_*t *_| *t *= 1, 2,..., *N*}, comprising the LS coefficients for all *N *trees obtained from pseudo-replicates was then computed:(3)

where *d*_*t *_(*i*, *j*) and δ_*t *_(*i*, *j*) are, respectively, the distance between the taxa *i *and *j *in the pseudo-replicated distance matrix **PRM**_*t *_and the patristic distance between the leaves labelled by *i *and *j *in the tree *t *inferred from **PRM**_*t *_(Figure 1). The maximum likelihood (ML) and maximum parsimony (MP) estimates can be used at this step as an alternative to LS. In the case of maximum parsimony, multiple optimal trees are usually generated for each replicate (note that multiple trees are possible with ML, although in practice they are not typically recovered). The resultant multiple pseudo-replicated trees can be treated in two following alternative ways: First, a consensus tree for these multiple trees can be established (e.g., using an extended majority rule) and then used in the computations in the same way that the unique NJ tree; second, each of the obtained multiple pseudo-replicated trees can directly contribute to the computation of the weighted bootstrap scores, but the resulting weights (Formulas 4-5) of each of those trees should be in turn divided by the cardinality of the set of optimal trees obtained for the considered set of pseudo-replicated sequences.

3. At this step the weights of all trees,**w **= {*w*_*t *_| *t *= 1, 2,..., *N*}, obtained from pseudo-replicates were computed by solving the following system of equations:(4)

The solution of the system (4) is as follows (for any *t *= 1,..., *N*):(5)

4. The LS-based bootstrap scores of the internal branches of *T*, denoted here as **ls**_**bs **= {*ls*_*bs*_*k *_| *k *= 1, 2,..., *m*}, were then determined. The LS-based bootstrap score of the branch *k *in *T *was computed as follows:(6)

where *m *is the number of internal branches of the original tree *T *and *B*_*t *_is the set of internal branches of the tree *t*.

### Normalized LS-based bootstrapping

Normalized LS-based bootstrap scores can also be computed and used to estimate the robustness of a phylogenetic tree. The normalization of LS, which should in most cases accentuate the difference between the LS coefficients associated with the phylogenetic trees inferred from pseudo-replicated data, was performed in the following way:(7)

where **norm**_**ls **= {*norm*_*ls*_*t *_| *t *= 1, 2,..., *N*} is the normalized vector of the least-squares coefficients computed after Step 2 (see the four-step weighting procedure described above) and *Min*(*ls*_1_, *ls*_2_,..., *ls*_*N*_) and *Max*(*ls*_1_, *ls*_2_,..., *ls*_*N*_) are, respectively, the minimal and maximal values of the set {*ls*_1_, *ls*_2_,..., *ls*_*N*_} computed at Step 2. Obviously, all the values of *norm_ls*_*t *_(*t *= 1, 2,..., *N*) are located in the [0,1] interval. Steps 3 and 4 were then carried out as described above using the normalized LS coefficients, and the weight of the tree *t *was computed as follows:(8)

### Secondary bootstrapping

**Figure 2:**
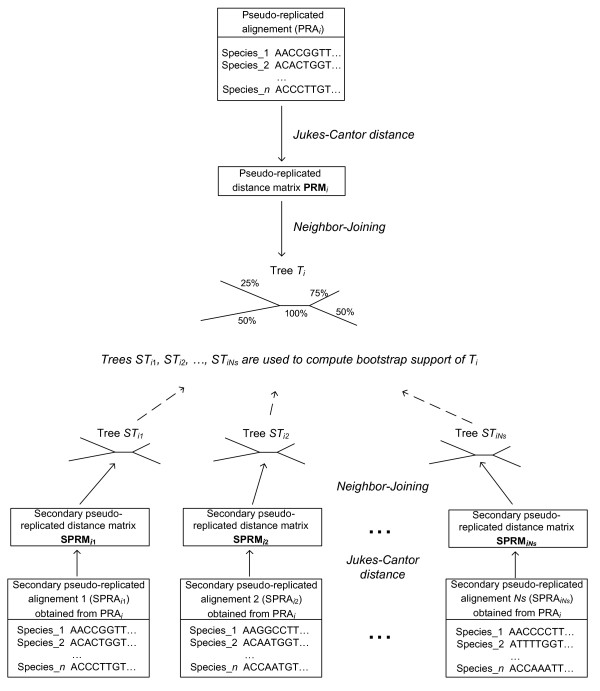
**Secondary bootstrapping**. Secondary bootstrapping: Secondary pseudo-replicated sequence alignments *SPRA*_*i*__1_, *SPRA*_*i*__2_,..., and *SPRA*_*iNs *_are obtained from the primary sequence alignment *PRA*_*i*_. The trees *T*_*i*__1_, *T*_*i*__2_,..., and *T*_*iNs *_inferred respectively from *SPRA*_*i*__1_, *SPRA*_*i*__2_,..., and *SPRA*_*iNs *_are serving to assess the quality of the phylogenetic tree *T*_*i *_used in primary (i.e., traditional) bootstrapping. A higher value of the average secondary bootstrap score of *T*_*i *_corresponds to its better (secondary) support and is associated with a higher weight.

Secondary bootstrap scores can be also used to assign weights to phylogenies inferred from pseudo-replicates. The weight of each phylogeny inferred from (primary) pseudo-replicated sequences can be assessed as the average of bootstrap scores of its internal branches. A pseudo-replicated sequence alignment *PRA*_*i *_(*i *= 1,..., *N*) can be used to create *Ns secondary *pseudo-replicated alignments *SPRA*_*i*__1_, *SPRA*_*i*__2_,..., *SPRA*_*iNs*_. As in traditional bootstrapping, the columns from *PRA*_*i *_can be randomly chosen with replacements to create *secondary pseudo-replicates*. Phylogenetic trees *T*_*i*__1_, *T*_*i*__2_,..., and *T*_*iNs *_can then be inferred from the pseudo-replicated alignments *SPRA*_*i*__1_, *SPRA*_*i*__2_,..., *SPRA*_*iNs*_, and the tree *T*_*i *_inferred from *PRA*_*i*_, using the same tree-building algorithm (Figure 2). The topology of *T*_*i *_can then be compared to the topologies of the trees built from the secondary pseudo-replicates. The bootstrap scores of all internal branches of *T*_*i *_can be computed, and the average bootstrap score (denoted here as *ss*_*i*_) characterizing the overall bootstrap support of the tree *T*_*i *_can be estimated.

When either the ML or MP approach is used, possible multiple optimal pseudo-replicated phylogenies can be treated in two ways: First, the mean of their average bootstrap scores can be taken into account in Formulas 9 and 10 and then their consensus tree in Formula 11; second, each of the obtained optimal MS or ML pseudo-replicated trees can directly contribute to the computation of the weighted bootstrap scores but their resulting weights (Formulas 9-10) should be divided by the cardinality of the set of optimal pseudo-replicated trees.

The weights **w **= {*w*_*t *_| *t *= 1, 2,..., *N*} of all the trees *T*_*i *_(*i *= 1,..., *N*) obtained from primary pseudo-replicates can be computed by solving the following equation system:(9)

The solution of the system (9) is as follows (for any *t *= 1,..., *N*):(10)

Obviously, the bigger the average secondary bootstrap score assigned to a tree, the bigger the tree weight.

The bootstrap scores of the internal branches of *T *based on secondary bootstrapping, and denoted as **ss_bs **= {*ss_bs*_*k *_| *k *= 1, 2,..., *m*}, can then be calculated. Thus, the bootstrap score of the branch *k *in *T *can be calculated as follows:(11)

where *m *is the number of internal branches of the original tree *T *and *B*_*t *_is the set of internal branches of the tree *t*.

### Normalized secondary bootstrapping

As in the case of the LS-based bootstrapping, the normalized secondary bootstrap scores can be computed and used to estimate the tree robustness. The normalization, which should emphasize the difference between the average secondary bootstrap scores of phylogenetic trees inferred from primary pseudo-replicates, can be carried out in the following way:(12)

where **norm_ss **= {*norm_ss*_*t *_| *t *= 1, 2,..., *N*} is the normalized vector of the average secondary bootstrap scores of primary pseudo-replicated trees *T*_1_,..., *T*_*N*_, and *Min*(*ss*_1_, *ss*_1_, .... *ss*_N_) and *Max*(*ss*_1_, *ss*_1_, .... *ss*_N_) are, respectively, the minimal and maximal values of the set {*ss*_1_,..., *ss*_*N*_}. Then, the weight of the primary pseudo-replicated tree *t *can be computed as follows:(13)

## Results

In this section we apply the four discussed weighting schemes to examine two real data sets consisting, first, of protein-coding mitochondrial DNA sequences for a group of 12 Primate species [15] and, second, of 32 PheRS Synthetase amino acid sequences for a group of 32 organisms, including bacteria, archaea and eukarya [16].

### Data description

The first examined data set was originally described by Hayasaka et al. [15]. The latter authors determined nucleotide sequences of homologous 896-base fragments of mitochondrial DNAs (mtDNAs) derived from four species of old-world monkeys, one species of new-world monkeys, two species of prosimians and five species of hominoids. They then reconstructed a phylogenetic tree for this group of 12 Primates. The internal branches of this tree have very high bootstrap support, varying from 85 to 100% (see the Results section). This data set was also analyzed in a number of evolutionary studies [20-23].

The second considered data set includes 32 PheRS Synthetase sequences with 171 bases for 21 bacteria, 6 archaea and 2 eukarya organisms originally studied by Woese et al. [16]. PheRS is the only class II synthetase in the NUN codon group, and it has no close relatives within that class. For both the α- and β-subunits of PheRS, significant length differences distinguish the bacterial subunits from their archaeal counterparts. Woese et al. [16] found that the AARSs were very informative about the evolutionary process. The analysis of different phylogenetic trees for a number of considered AARSs revealed the following features: The AARSs evolutionary relationships were mostly conform to established species phylogeny; a strong distinction existed between bacterial and archaeal types of AARSs; horizontal transfer of AARS genes between archaea and bacteria was predicted (see also [24]). In fact, PheRS shows classical canonical pattern with the only exception being the spirochetes (i.e., *Borrelia burgdorferi *and *Treponema pallidum*) PheRSs. They are of the archaeal, not the bacterial genre, and are closely related to the clade formed by the archaea *Pyrococcus horikoshii*, *Pyrobaculum aerophilum *and *Sulfolobus solfataricus *(see the Results section and Figure two in [16]). The considered PheRS data set was also studied intensively [24-33].

### Distribution of the LS coefficients and average secondary bootstrap scores

**Figure 3:**
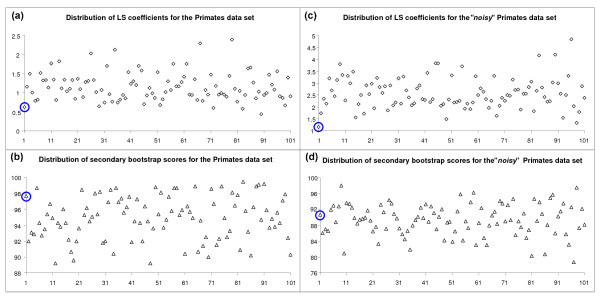
**Distribution of the LS coefficients and SBS for the Primate data set**. Distribution of the LS coefficients for the original (a) and "noisy" (b) Primate [15] multiple sequence alignments (MSAs) and 100 pseudo-replicated data sets obtained from each of them. Distribution of the average secondary bootstrap scores of the trees corresponding to the original (c) and "noisy" (d) Primate MSAs and 100 pseudo-replicated data sets obtained from each of them. The first (encircled) value corresponds to the original (cases a and b) and noise-affected original (cases c and d) MSAs.

**Figure 4:**
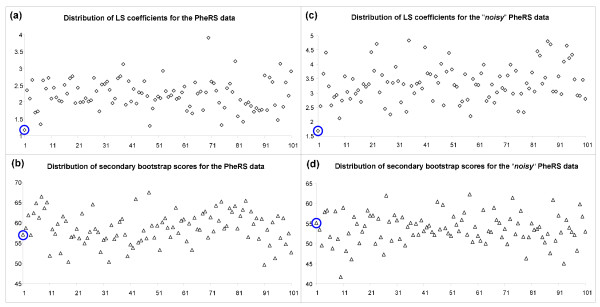
**Distribution of the LS coefficients and SBS for the PheRS data set**. Distribution of the LS coefficients for the original (a) and "noisy" (b) PheRS [16] multiple sequence alignments (MSAs) and 100 pseudo-replicated data sets obtained from each of them. Distribution of the average secondary bootstrap scores of the trees corresponding to the original (c) and "noisy" (d) PheRS MSAs and 100 pseudo-replicated data sets obtained from each of them. The first (encircled) value corresponds to the original (cases a and b) and noise-affected original (cases c and d) MSAs.

First, we examined the distribution of the least-squares (LS) coefficients and secondary bootstrap scores (SBS) for the Primate [15] and PheRS Synthetase [16] data sets presented above (Figures 3 and 4, cases a-b). For both original multiple sequence alignments (MSA), we also created their "noisy" variants by modifying 10% of the nucleotides for the Primate MSA and amino acids for the PheRS MSA (Figures 3 and 4, cases c-d). The noise-affected data were generated in order to investigate how the LS and SBS functions change when the uncertainty is introduced in the data. Figures 3 and 4 show the distribution of LS and SBS for the original (a) and "noisy" (b) MSAs as well as for 100 pseudo-replicated data sets obtained from each of them.

Figures 3a and 4a show that the LS coefficients corresponding to the original MSAs (depicted by encircled diamonds in both figures) are very low (e.g., the lowest LS coefficient in Figure 4a is that of the original MSA). This means that the original MSAs were generally much closer to the space of phylogenetic trees than the pseudo-replicated MSAs obtained from them. After the addition of noise (Figures 3c and 4c) the LS coefficients corresponding to the original and pseudo-replicated MSAs obviously increased, but the difference between them emphasized: The LS coefficient of both original trees (Figures 3c and 4c) became the smallest ones in both cases.

On the other hand, the average SBS corresponding to the original trees (see the encircled triangles in Figures 3b and 4b) were not among the highest ones compared to those of the "pseudo-replicated" trees. This means that the original trees were not necessarily more robust than their pseudo-replicated counterparts. After the addition of noise (Figures 3d and 4d), the robustness of the original and "pseudo-replicated" trees decreased as expected. For the noisy data, the average SBS of the original trees remained only slightly higher than the mean of the average SBS of the "pseudo-replicated" trees.

### Simulation study

A simulation study was conducted to evaluate the performances of the four introduced weighting strategies, including the LS and SBS-based (original and normalized) bootstrapping. The traditional bootstrapping scheme, assigning the weights of 1 to all pseudo-replicated trees, was also tested. The simulations were carried out on the Primate [15] and PheRS Synthetase [16] data sets discussed above.

In order to examine the robustness of each weighting strategy, a simulation with "noisy" sequences was performed. A random noise varying from 1 to 10% (with the step of 1%) was added to both original MSAs (for the Primate and PheRS data) to create the variants of "noisy" data. To simulate noisy data in the aligned sequences, we tested two experimental strategies. The first strategy consisted of changing at random a fixed percentage of nucleotides from the observed sequence, whereas the second one consisted of the random elimination or addition of blocks of nucleotides (or amino acids) of different sizes. In this section, we are presenting the combined results (with the 50/50% ratio) for these two strategies detailed below.

*Strategy *1. For a given noise percentage (*NR*%), each nucleotide or amino acid of the original data set had the probability of *NR*% to change its state. If the nucleotide or amino acid *x *was chosen to be affected by noise, it was replaced by a different nucleotide or amino acid. All the other nucleotides or amino acids, different from *x*, had an equal probability (1/3 for nucleotides and 1/19 for amino acids) to replace *x *in the MSA. The sequences were not realigned after the addition of noise.

*Strategy *2. For a given percentage of noise (*NR*%), the random elimination or addition of blocks of nucleotides (or amino acids) of different sizes (the block sizes were selected randomly and varied from n*l*NR/2 to n*l*NR/10 nucleotides or amino acids, where *n *was the number of species and *l *was the sequence length) was performed. The elimination of blocks of nucleotides or amino acids imitates possible deletion events and introduces new gaps in the multiple sequence alignment. The addition of short sequences of nucleotides or amino acids imitates possible insertion events.

The Seqboot program from the PHYLIP package [34] was used to generate multiple resampled versions of the original Primate and PheRS MSA. For each execution, 100 replicates of the original data sets were generated. All the other parameters used were the default Seqboot parameters. The Jukes-Cantor [18], in the case of nucleotides, and Kimura Protein [19], in the case of amino acids, sequence-to-distance transformations followed by the Neighbor-Joining algorithm [17] were carried out to infer phylogenetic trees. The five bootstrapping strategies (4 relying of weights and the traditional one) were tested on such noisy pseudo-replicates. For each of the five strategies, the following measure, denoted here as *least-squares bootstrap deviation *- *ls_bd*, was calculated as follows to assess the strategy robustness:(14)

where *bs*_*k *_is the bootstrap score of the internal branch *k *in the original tree *T *inferred from the original MSA (i.e., from the original Primate or PheRS data set), *bsn*_*k *_is the bootstrap score of the internal branch *k *in the tree *T*_*noisy *_obtained from the original MSA affected by noise, and *m *is the number of internal branches in the original tree *T *(note that *m *was always equal to *n*-3, where *n *was the number of species, for both Primate and PheRS phylogenies).

**Figure 5:**
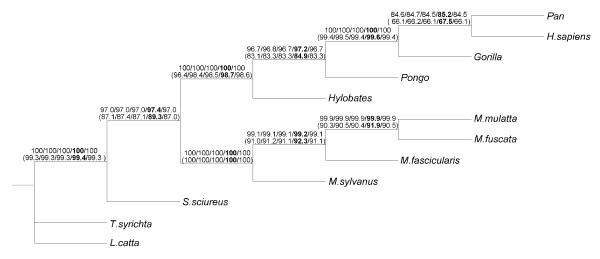
**Primate phylogenetic tree with bootstrap scores**. The 12-taxon Primate phylogenetic tree inferred with Neighbor-Joining [17]. The nucleotide sequences of 896-base fragments of mitochondrial DNAs [15] were considered and transformed into distances using the Jukes-Cantor transformation [18]. The bootstrap scores provided by the five considered bootstrapping strategies are indicated above internal branches. They are shown for the original and noisy data (the noisy data were obtained after the addition of 10% of noise to the original sequences; they are indicated between parentheses). The bootstrap scores are indicated in the following order: Standard bootstrap scores, secondary bootstrap scores, LS-based bootstrap scores, normalized secondary bootstrap scores (shown in bold) and normalized LS-based bootstrap scores.

**Figure 6:**
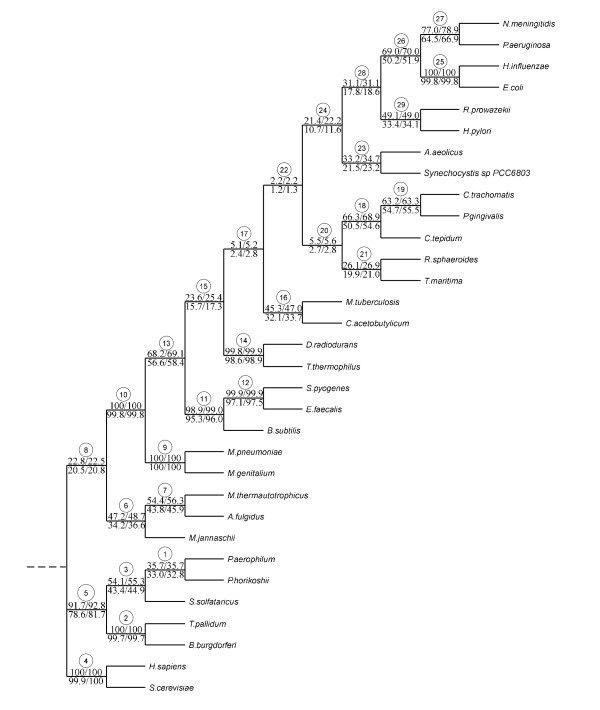
**PheRS phylogenetic tree with bootstrap scores**. The 32-taxon PheRS Synthetase [16] phylogeny inferred using Neighbor-Joining [17]. The standard bootstrap scores, followed by normalized secondary bootstrap scores, are indicated above the internal branches. They are shown for the original and noisy data (the noisy data were obtained after the addition of 10% of noise to the original sequences; they are indicated under the internal branches). The bootstrap scores for the three other weighting bootstrap strategies are presented in Table 1 (encircled, are the branch numbers as they are reported in Table 1).

Figures 5 and 6 report, respectively, the Primate [15] and PheRS [16] phylogenies built with Neighbor-Joining. It is worth noting that the Primate phylogenetic tree (Figure 5) perfectly corresponds to that previously obtained by Makarenkov and Legendre [21], whereas the PheRS phylogeny (Figure 6) was different from the tree obtained by Woese et al. [16] and Boc et al. [24], using the ML methods. The most noticeable difference between the presented NJ phylogeny (Figure 6) and the ML trees built by Woese et al. [16] and Boc et al. [24] is that in the tree in Figure 6 the spirochetes (i.e., PheRSs of the bacteria *B. burgdorferi *and *T. pallidum*) are not specifically related to the archaebacterium *P. horikoshii *(these three organisms form a 3-taxon cluster in the trees shown in Figure two in [16] and Figure seven in [24]). The bootstrap scores provided by the five competing bootstrapping strategies (i.e., traditional bootstrap scores, secondary bootstrap scores, LS-based bootstrap scores, normalized secondary bootstrap scores and normalized LS-based bootstrap scores) were calculated for the original and noisy data and depicted in Figure 5 (for the Primate data) and Figure 6 and Table 1 (for the PheRS data). The results presented in Figures 5 and 6, and in Table 1 demonstrate that the normalized secondary bootstrap scores were usually higher than the bootstrap scores yielded by the four other bootstrapping strategies, including the traditional bootstrapping method. This trend was maintained for both original and noisy data. On the other hand, the bootstrap scores provided by the secondary bootstrapping, LS-based bootstrapping, and normalized LS-based bootstrapping were very similar to those obtained with the traditional unweighted bootstrapping. For instance for the original (and, respectively, for the noisy) PheRS data, the standard bootstrap scores were lower than those given by the normalized secondary bootstrap scores strategy in 18 of 29 cases (24 of 29 cases for the noisy data), equal in 9 cases (4 cases for the noisy data) and higher in only 2 cases (1 case for the noisy data). Thus, when a 10%-noise was added to the data, the difference in the bootstrap scores even emphasized. The indicated scores for the original and noisy data, for each of the tested noise percentages, were the averages calculated over 100 repeated calculations (for both primary and secondary bootstrapping).

**Table 1:** Bootstrap scores comparison for the PheRS data set

*Branch number*	*Scores for noise-free data*					*Scores for noisy data (10% of noise)*				
	
	Std	SB	LS	NSB	NLS	Std	SB	LS	NSB	NLS
1	35.7	35.7	35.4	**35.7**	35.2	33.0	33.0	32.6	**32.8**	32.4
2	100.0	100.0	100.0	**100.0**	100.0	99.7	99.7	99.7	**99.7**	99.7
3	54.1	54.3	53.7	**55.3**	53.4	43.4	43.6	43.0	**44.9**	42.8
4	100.0	100.0	100.0	**100.0**	100.0	99.9	99.9	99.9	**100.0**	99.9
5	91.7	91.9	91.8	**92.8**	91.7	78.6	79.2	78.7	**81.7**	78.7
6	47.2	47.4	47.6	**48.7**	47.9	34.2	34.6	34.6	**36.6**	34.8
7	54.4	54.7	54.4	**56.3**	54.5	43.8	44.2	43.8	**45.9**	43.8
8	22.8	22.7	22.7	**22.5**	22.7	20.5	20.5	20.4	**20.8**	20.3
9	100.0	100.0	100.0	**100.0**	100.0	100.0	100.0	100.0	**100.0**	100.0
10	100.0	100.0	100.0	**100.0**	100.0	99.8	99.8	99.8	**99.8**	99.8
11	98.9	98.9	99.0	**99.0**	99.0	95.3	95.4	95.5	**96.0**	95.6
12	99.9	99.9	99.9	**99.9**	99.9	97.1	97.2	97.0	**97.5**	97.0
13	68.2	68.3	68.8	**69.1**	69.2	56.6	56.9	57.6	**58.4**	58.3
14	99.8	99.8	99.8	**99.9**	99.9	98.6	98.7	98.7	**98.9**	98.7
15	23.6	23.9	23.5	**25.4**	23.5	15.7	16.0	15.8	**17.3**	16.0
16	45.3	45.6	45.5	**47.0**	45.6	32.1	32.4	32.4	**33.7**	32.4
17	5.1	5.1	5.0	**5.2**	5.0	2.4	2.5	2.5	**2.8**	2.5
18	66.3	66.8	67.0	**68.9**	67.4	50.5	51.2	51.0	**54.6**	51.4
19	63.2	63.2	63.5	**63.3**	63.7	54.7	54.9	55.0	**55.5**	55.3
20	5.5	5.5	5.6	**5.6**	5.7	2.7	2.7	2.7	**2.8**	2.7
21	26.1	26.2	26.1	**26.9**	26.2	19.9	20.1	20.0	**21.0**	20.0
22	2.2	2.2	2.2	**2.2**	2.3	1.2	1.2	1.2	**1.3**	1.3
23	33.2	33.5	32.9	**34.7**	32.7	21.5	21.8	21.2	**23.2**	21.1
24	21.4	21.5	21.2	**22.2**	21.2	10.7	10.8	10.6	**11.6**	10.7
25	100.0	100.0	100.0	**100.0**	100.0	99.8	99.8	99.7	**99.8**	99.7
26	69.0	69.2	69.4	**70.0**	69.5	50.2	50.5	50.4	**51.9**	50.6
27	77.0	77.3	76.9	**78.9**	77.0	64.5	64.9	64.5	**66.9**	64.6
28	31.1	31.1	31.0	**31.1**	30.9	17.8	17.9	17.8	**18.6**	17.9
29	49.1	49.1	49.3	**49.0**	49.3	33.4	33.5	33.5	**34.1**	33.6

Comparison of bootstrap scores for the five bootstrapping strategies considered in this study. The comparison was made for all 32 internal branches of the phylogenetic trees inferred from the original (i.e., noise-free) and noisy (with 10% of noise added) PheRS sequences. The branch numbers correspond to those indicated in Figure 6. Standard bootstrap scores (Std), secondary bootstrap scores (SB), LS-based bootstrap scores (LS), normalized secondary bootstrap scores (NSB, shown in bold) and normalized LS-based bootstrap scores (NLS) are reported.

**Figure 7:**
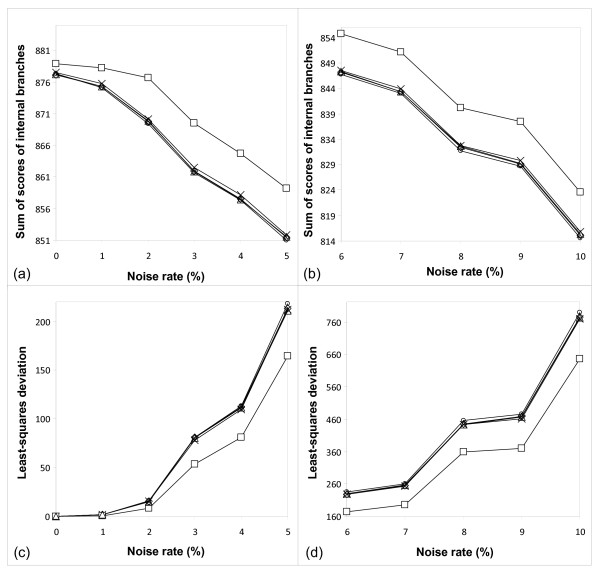
**Sum of bootstrap scores and LS deviation for the Primate data set**. Sum of bootstrap scores of internal branches (cases a and b) and least-squares bootstrap deviation (cases c and d), computed according to Formula 14, for the Primate data set [15] obtained by the five bootstrapping strategies considered in this study. The results are shown with respect to the noise level (the noise percentage varying from 1 to 10% is represented on the *x*-axis). Standard bootstrap scores are depicted by circles, secondary bootstrap scores by crosses, LS-based bootstrap scores by diamonds, normalized secondary bootstrap scores by squares, and normalized LS-based bootstrap scores by triangles.

**Figure 8:**
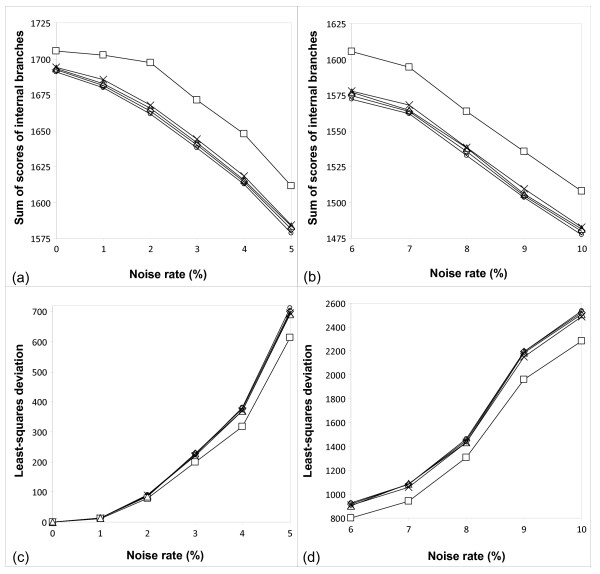
**Sum of bootstrap scores and LS deviation for the PheRS data set**. Sum of bootstrap scores of internal branches (cases a and b) and least-squares bootstrap deviation (cases c and d), computed according to Formula 14, for the PheRS Synthetase data set [16] obtained by the five bootstrapping strategies considered in this study. The results are shown with respect to the noise level (the noise percentage varying from 1 to 10% is represented on the *x*-axis). Standard bootstrap scores are depicted by circles, secondary bootstrap scores by crosses, LS-based bootstrap scores by diamonds, normalized secondary bootstrap scores by squares, and normalized LS-based bootstrap scores by triangles.

Moreover, Figures 7 and 8, representing, respectively, the Primate [15] and PheRS [16] data, illustrate the difference in the following parameters between the five bootstrapping strategies: Sum of bootstrap scores of internal branches (Figures 7-8 a-b) and least-squares bootstrap deviation (Figures 7-8 c-d). The latter parameter, computed according to Formula 14, can be viewed as an indicator of the method's robustness. Indeed, the lower the method sensitivity regarding the noise factor, the smaller the least-squares bootstrap deviation. The results in Figures 7-8 are shown depending on the noise percentage (varying from 1 to 10%). When observing the sum of bootstrap scores and the least-squares bootstrap deviation curves, one can notice that the normalized secondary bootstrap scores strategy always provided the highest totals of bootstrap scores of internal branches and the lowest least-squares bootstrap deviations regardless the noise level. For instance for the Primate data set and the normalized secondary bootstrapping, the least-squares bootstrap deviation, *ls_bd*, between the noise-free and noisy bootstrap scores (Formula 14) was equal to 644.01, while for the traditional bootstrapping, the *ls_bd *coefficient was much higher and equal to 786.4. Alternatively, for the PheRS data set and the normalized secondary bootstrapping, the *ls_bd *coefficient was equal to 2279.19, while for the traditional bootstrapping it was also much higher and equal to 2534.58. The additional simulations conducted with larger noise levels (when the noise factor varied from 10 to 35%; these results are not shown) confirmed the observed trend.

## Discussion

The controversial study conducted by Hillis and Bull [35] claimed that the traditional bootstrap confidence values used to assess tree accuracy are consistently biased downward. As a response to Hillis and Bull [35], Felsenstein and Kishino [36] argued that the phenomena noticed in [35] are not the result of bootstrap use but rather a result of summarizing the evidence for a given clade using the associated p-values.

Later on, Efron et al. [37] introduced a method for bias correction to estimate more accurate p-values for topological inference through a correction based on first-order p-values. The simplex of possible solutions is partitioned into regions corresponding to different tree topologies [37,38]. Efron's study concluded that the confidence values  obtained using the traditional Felsenstein's bootstrapping are not systematically conservative (i.e., not biased systematically downward) as was stated by Bull and Hillis [35]. Depending on the local configuration of the topological space around the actual tree, the bias may be conservative or liberal. According to Efron's study [37], Felsenstein's method provides a reasonable first approximation to the actual confidence levels of the observed tree clades. One interpretation of non-parametric bootstrapping that is compatible with Bayesian inference was also proposed [[37], page 7090]: "In a Bayesian sense,  can be thought of as reasonable assessments of error". Efron et al. [37] defined another type of non-Bayesian confidence level  (which can be estimated by a two-level bootstrap algorithm), such that  and  converge at rate , as the sequence length *n *increases. The methods discussed in [37] and [38] assess the curvature of the solution boundary, which is used in an analytical correction formula to estimate the magnitude of the shifted bootstrap distribution.

In [38], Efron and Tibshirani introduced the "problem of regions". There, one wishes to know which of a discrete set of possibilities applies to a continuous parameter vector. Efron and Tibshirani gave several examples of problem of regions that appear in real applications, including testing significance for model selection and for the number of density peaks. They concluded that, at some point, third-order and higher terms may be necessary to obtain sufficiently accurate confidence estimates [38]. Both of the latter studies used weighting procedures. However, the weights described in [[37] and [38]] are not applied to pseudo-replicated trees, as in our study, but to the first-level bootstrap vectors. The procedure of reweighting the first-order resamples is carried out using a *simple importance sampling scheme *[see the Bootstrap reweighting section in 38 and Equations 4.1-4.14 therein]. According to [38], reweighting the first-order bootstrap samples converts, from a Bayesian point of view, the flat-prior of *a posteriori *probability distribution of the related regions into the appropriate Welch-Peers *a posteriori *probabilities.

Furthermore, the method introduced in this paper is based on the *quality *of pseudo-replicated trees (expressed through the LS and SBS measures) used in the classical Felsenstein's bootstrapping, whereas the Efron method [37], based on an iterative bootstrapping, searches directly for the improvement of the bootstrap scores robustness. It is worth noting that the second-level bootstrap vectors considered in [37] (that are somewhat analogous to the SBS considered in this paper) are generated only for the first-level bootstrap vectors *located on the boundary *of the clade whose robustness is evaluated.

In their recent work, Gullo et al. [39] discussed the usage of different weighting schemes for clustering ensembles, including the problems of consensus tree reconstruction and bootstrap support computation. Clustering ensembles (i.e., consensus clustering or aggregation clustering) have recently emerged as a powerful tool to address traditional clustering issues [39]. Given a data collection, a set of clustering solutions (i.e., ensemble), can be generated by varying the parameter settings. Given a clustering ensemble (in our case, the set of trees obtained from resampled sequences), a major goal is to extract a consensus partition (in our case, the original tree with a robust bootstrap support), taking into account information available from the given set of clustering solutions. Gullo et al. [39] provided the justification for several weighting schemes to discriminate among the clustering solutions, including the one adopted in the present study. Each of these schemes is based on theoretical considerations on ensemble diversity and computes the vector of weights **w **= (*w*_1_,..., *w*_*N*_) in such a way that *w*_*i *_∈ [0; 1], for each *i *∈ [1,...,*N*], and . The first of those schemes, called *Single Weighting *(see Formula 4.4 in [39]), presents the most intuitive way to weight each clustering solution (i.e., each pseudo-replicated tree in our case). The key idea consists in computing each individual cluster diversity measure (in our case, such a measure would be the LS coefficient or the average SBS associated with each pseudo-replicated phylogeny) and then in assigning weights that are *proportional *to individual cluster diversities. In fact, Formulas 5 and 10 used in our study to determine the individual weights of pseudo-replicated trees are analogous to Formula 4.4 in [39]. These formulas represent the simplest and the most intuitive way of introducing weights in bootstrap analysis. Most research works focusing on clustering ensembles diversity suggest selecting ensembles according to a maximum diversity criterion [40-42], which states that the higher the ensemble diversity (i.e., the more variation we have in the individual LS coefficients or in the average SBS), the better the accuracy of the consensus partition (i.e., bootstrap scores or consensus tree) extracted from the ensemble. Thus, in our study, the weights are computed using a linearly increasing distribution, which defines weights according to a maximum diversity criterion. In the future, it would be also interesting to test the other weighting schemes discussed in [39]. Specifically, a Normal distribution model that computes weights according to a median diversity criterion (see Formula 4.5 in [39]) along with the Group Weighting (see Formulas 4.6-4.9 in [39]) and Dendrogram Weighting (see Formula 4.10 and Algorithm 1 in [39]) models could be tested in the framework of weighted bootstrapping.

## Conclusions

The traditional non-parametric bootstrapping is a common method for assessing tree confidence in phylogenetic analysis [2]. It generates and operates pseudo-replicated (i.e., resampled) data sets having the same empirical distribution that the original data set. However, traditional bootstrapping does not take into account either the "tree-likeness" of phylogenies inferred from pseudo-replicated sequences (i.e., how well these phylogenies fit the corresponding pseudo-replicated sets of sequences) or the bootstrap support of those phylogenies. In this study, we described four weighting strategies allowing one to assign weights to the trees inferred from pseudo-replicates, and thus to do away with one of the limitations of traditional bootstrapping: The assignment of equal weights to all "pseudo-replicated" trees. In our approach, the weights of the trees inferred from pseudo-replicates are assigned according to either the LS estimate of this tree (i.e., how well it fits the pseudo-replicated sequences) or to the average secondary bootstrap scores (SBS) of the tree (i.e., the bootstrap scores associated with the internal branches of "pseudo-replicated" trees). The simulations carried out with two real data sets and five weighting strategies, including the LS and SBS-based bootstrapping, the LS and SBS-based bootstrapping with the data normalization, and the traditional bootstrapping, suggest that the weighted bootstrapping based on the normalized SBS usually exhibits larger bootstrap scores and a higher robustness compared to the traditional bootstrapping and the three other competing methods. The high robustness of the weighting strategy based on the normalized SBS makes this strategy particularly useful in the situations when the considered sequences were affected by noise or underwent insertion or deletion events. Also, when large numbers of replicates (≥100) were considered, the performances of the four other weighting strategies were very similar, thus confirming the stability of the traditional unweighted bootstrapping.

An interesting way for the future investigation would be the study of the proposed weighting schemes in the context of establishing a consensus tree. For instance, the *Consense *program of the PHYLIP package [34] allows the user to introduce weights for each of the input trees. Indeed, the average SBS or LS (original or normalized) estimates of the trees (e.g., of the trees obtained from the pseudo-replicated sequences) could be used to compute the consensus tree (see also [43]). In addition, a new way of computing a consensus tree, which takes into account all individual bootstrap scores of the internal branches of the input trees, could be developed for the weighted supertree methods discussed in [44].

## Authors' contributions

VM, AB and JX designed the methods, implemented them and carried out the simulations. VM, PP-N, F-JL and PL supervised the project and coordinated the development of the methods. All authors read and approved the final manuscript.
